# Physical properties and mass models of Deglet Noor and Arichti semi‐dry Algerian date fruits: A comparative study

**DOI:** 10.1002/fsn3.3969

**Published:** 2024-02-15

**Authors:** Messaoud Roumani, Rania Remmani, Malek Miladi, Nawaf Abu‐Khalaf, Antonio Ruiz Canales

**Affiliations:** ^1^ Department of Phœniciculture Scientific and Technical Research Center on Arid Regions Biskra Algeria; ^2^ Applied Chemistry Laboratory University of Biskra Biskra Algeria; ^3^ Engineering Department Miguel Hernández University Alicante Spain; ^4^ Department of Agricultural Biotechnology Palestine Technical University‐Kadoorie Tulkarm Palestine

**Keywords:** Algerian date varieties, arid and semi‐arid regions, date fruit, mass modeling, physical properties

## Abstract

For thousands of years, date fruit (*Phoenix dactylifera* L.) has been a popular diet in arid and semi‐arid locations. It has religious importance for Muslims and is especially important during the holy month of Ramadan. The global output of date fruits has been continuously expanding, with Arab nations accounting for a sizable portion. The emphasis of this research is on two popular semi‐dry Algerian date fruit types, Deglet Noor and Arechti, which are grown in the Ziban region. These fruits' physical parameters, such as size, sphericity, surface area, volumes, and density, were determined. The goal of this study was to create a mass model based on these physical attributes to help in the grading and sorting of date fruits. Fruit mass was shown to be closely connected to linear dimensions, arithmetic and geometric mean diameters, surface area, and volumes. Correlations between mass and physical attributes were established using a variety of mathematical models, including linear, quadratic, S‐curve, and power models. The results demonstrated the applicability of specific factors for mass modeling, offering useful insights for the development of system sizing and conservation. With good correlation, multivariate data analysis was employed to correctly estimate the mass of both kinds. This research advances our understanding of the physical features of Algerian date fruits and their connection to mass, allowing for better handling, sorting, and packing processes in the worldwide date market.

## Graphical abstract

This collaborative investigation systematically elucidates the nuanced physical properties of Deglet Noor and Arichti Algerian date fruits, encompassing comprehensive assessments of dimensions, surface area, and volumes. The study meticulously formulates robust mass models, imperative for precise sorting and packaging methodologies. The resultant comparative analysis augments scholarly comprehension of these date cultivars, thereby substantively enriching the corpus of knowledge within the domain of food science and nutritional practices.

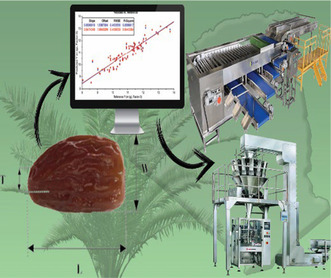

## 1 INTRODUCTION

Date fruit (*Phoenix dactylifera* L.) has been a fundamental food source for over 6000 years, especially in arid and semi‐arid regions like the Arab countries in the Middle East and North Africa (Ashraf and Hamidi‐Esfahani, 2011; Khwaldia et al., 2023). These areas face challenging climatic conditions, including high temperatures, limited precipitation, and arid soils, which pose significant obstacles for the growth and development of many edible plant species (Al Juhaimi et al., 2014; Tang et al., 2013). Despite these challenges, the date palm has proven to be a remarkable crop, exhibiting exceptional resilience and adaptability to adverse environments, providing sustenance to local populations for millennia (Alotaibi et al., 2023).

The cultural and religious significance of date fruits, especially within the Muslim community, cannot be overstated. The Holy Quran contains numerous references to the date palm, elevating its status as a revered and symbolic tree (Al‐Farsi & Lee, 2008). Notably, dates hold a prominent place in religious practices, particularly during the holy month of Ramadan. The consumption of dates plays a central role in the iftar meal, marking the end of the day‐long fast for individuals who have abstained from food and drink throughout daylight hours (Al‐Shahib & Marshall, 2003; Baliga et al., 2011). This practice not only fulfills the physiological needs of fasting individuals but also holds profound spiritual and communal significance, fostering a sense of unity, gratitude, and shared experience (Al‐Farsi & Lee, 2008; Khwaldia et al., 2023).

Given the historical and cultural contexts, it becomes evident that date fruits play a pivotal role in the sustenance, traditions, and cultural identity of communities residing in arid and semi‐arid regions (Mohamed et al., 2014; Muñoz‐Bas et al., 2023). Furthermore, the global demand for date fruits has experienced a notable surge in recent years. The production of dates has more than doubled since 1994, with recorded global output reaching approximately 8.50 million tons in 2016 (Altaheri & Alsulaiman, 2019). Arab countries, benefiting from favorable climatic conditions conducive to date palm cultivation, make a substantial contribution to the world's date production, accounting for an impressive 91% of the total output (Altaheri & Alsulaiman, 2019).

Given the rising need and the paramount importance of date fruits, it becomes imperative to gain a comprehensive understanding of their inherent characteristics, remarkable diversity, and potential applications. Notably, exploring the genetic variability among date palms in North Africa and the Middle East has emerged as a focal point in recent research endeavors. Numerous studies have documented the existence of over 3000 distinct date palm varieties, each exhibiting unique traits and attributes (Battesti et al., 2018). Among the well‐known commercial varieties that have gained popularity due to their exceptional taste, texture, and culinary versatility are Khalas, Medjool, and Deglet Nour (Al‐Mssallem et al., 2013; Battesti et al., 2018; Hazzouri et al., 2015). By delving into the genetic makeup and distinct properties of these varieties, researchers can unlock valuable insights into their agricultural potential, nutritional composition, and utilization in various culinary and industrial applications (Hazzouri et al., 2019).

Date palm cultivars are classified based on the texture of their flesh, resulting in three distinct groups: soft, dry, and semi‐dry (Biglari et al., 2008; Emam et al., 1994). Among the semi‐dry varieties, Deglet Noor cv. and Arechti cv. prominently thrive in the Ziban region of Algeria, specifically within the Biskra Province (Hameurlaine et al., 2018).

Deglet Noor fruits exhibit an ovoid morphology and showcase a spectrum of hues ranging from reddish to yellow (Ghnimi et al., 2017). The flesh of Deglet Noor is characterized by its remarkable transparency, tenderness, fibrous consistency, and delicately perfumed flavor profile (Hannachi et al., 1998). Cultivation and production of Deglet Noor predominantly transpire within Algeria and Tunisia, collectively accounting for approximately 90% of the global output (Amroussia et al., 2023). Notably, Algeria concentrates its cultivation efforts in three pivotal regions, namely Ziban, Oued Souf, and M'zab (Amziane, 2016; Bouguedoura et al., 2015).

In stark contrast to Deglet Noor, Arechti cultivars manifest distinct attributes worthy of exploration. Arechti fruits display a distinctive brown coloration and exhibit an ovoid shape, setting them apart from their counterparts (Simozrag and Laiadi, 2020). The flesh of Arechti showcases commendable tenderness and accompanies an enticing acidulous taste sensation (Hannachi et al., 1998). In Algeria, the primary production of Arechti is primarily concentrated in the Ziban and Oued Souf regions, making a significant contribution to the local agricultural landscape (Bouguedoura et al., 2015).

The contrasting characteristics between Deglet Noor and Arechti cultivars offer an intriguing avenue to investigate the diverse qualities and culinary applications inherent in different date palm varieties prevalent in the Ziban region of Algeria. Delving deeper into the unique attributes of each cultivar holds the potential to provide valuable insights into their agronomic potential, nutritional composition, and prospective utilization across various culinary and industrial domains (Mohsenin, 2020).

A comprehensive understanding of the physical properties and interrelationships of date palm fruits is crucial for the global date market, particularly in handling, sorting, processing, and packaging (Hayyan et al., 2012; El Arem et al., 2012). The design of efficient grading systems must consider dimensions, weight, and volume, as these factors directly influence consumer preferences for uniformly sized and shaped fruits and vegetables (Afshari et al., 2023). Mass grading methodologies not only lead to cost savings in packaging and shipping but also facilitate optimal packaging arrangements (Mahawar et al., 2019).

The significance of these efforts is amplified within the global date market, where meeting consumer demands efficiently and with a focus on quality is imperative (Benziouche & Cheriet, 2012; Jaliliantabar & Lorestani, 2014; Shahbazi & Rahmati, 2013). Implementing mass grading techniques in the evaluation of vegetables and fruits offers notable advantages in the commercial landscape. By classifying produce based on physical attributes such as dimensions, weight, and volume, producers and distributors can streamline operations and optimize supply chain efficiency. Standardized grading systems address consumer preferences for uniformity while promoting resource optimization, including packaging materials and transportation logistics. This results in cost savings and advances sustainable practices within the date fruit industry.

Moreover, the integration of precise grading methodologies empowers market participants to maintain a competitive edge by consistently delivering high‐quality products aligned with consumer expectations. Consequently, the exploration and application of innovative grading approaches remain pivotal to the continued growth and prosperity of the global date market (Afshari et al., 2023; Wilhelm et al., 2004).

Until recently, detailed studies on date fruit mass modeling in the South Algerian zone were lacking. This study aims to fill this gap by conducting a thorough analysis and comparative assessment of the physical characteristics and mass fluctuations exhibited by Algerian Deglet Noor and Arechti semi‐dry date fruits. The originality of this research lies in its comprehensive examination of these widely consumed date varieties, with a specific focus on their morphological attributes and weight variations. Through precise measurements and meticulous evaluations, this study provides valuable insights into the divergences observed in terms of size, shape, texture, and mass of these fruits. Such an examination contributes to an enhanced understanding of their intrinsic quality and potential applications. The findings of this investigation carry practical implications for date cultivators, processors, and consumers, empowering them to make well‐informed decisions regarding date selection, storage, and utilization strategies.

## 2 MATERIALS AND METHODS

### 2.1 Date samples

**FIGURE 1:**
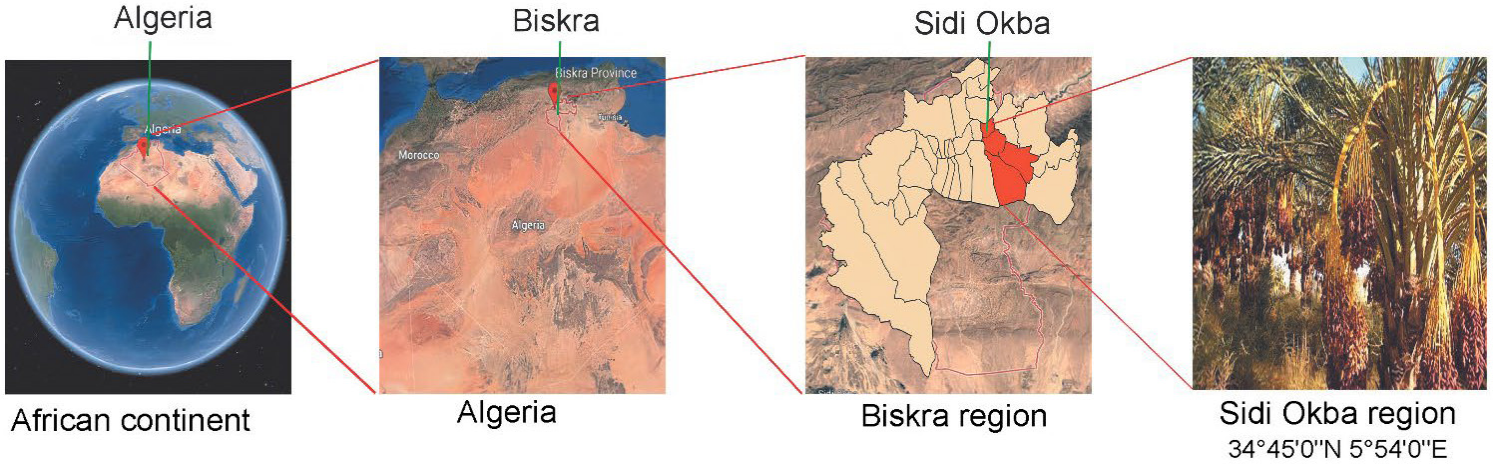
Location map of the study area.

The fruit samples under investigation were randomly collected from local date fruit gardens in the Sidi Okba region of Biskra Province (470 km southeast of Algiers, Algeria; Figure 1). Subsequently, the date fruit samples were washed and dried before being packed in polyethylene bags at 5°C until use. Prior to the start of the experiments, the samples were allowed to reach room temperature.

### 2.2 Physical properties

Within the same variety of fruit, randomly chosen dates typically exhibit varying physical characteristics (Li et al., 2011). The determination of these properties plays a crucial role in improving the design and efficiency of equipment for harvesting, transporting, cleaning, packing, storing, and processing the fruit (Kabas et al., 2006; Kiliçkan & Güner, 2008; Li et al., 2011; Rahemi et al., 2020). Numerous recent studies have delved into the physical properties of date fruits, exploring varieties such as Algerian Mech‐Degla and red cultivars (Djouab et al., 2016), Tunisian Kentichi cultivars (Herch et al., 2014), and Iranian Dairi cultivars (Jahromi et al., 2008).

#### 2.2.1 Dimensions of date palm fruits

**FIGURE 2:**
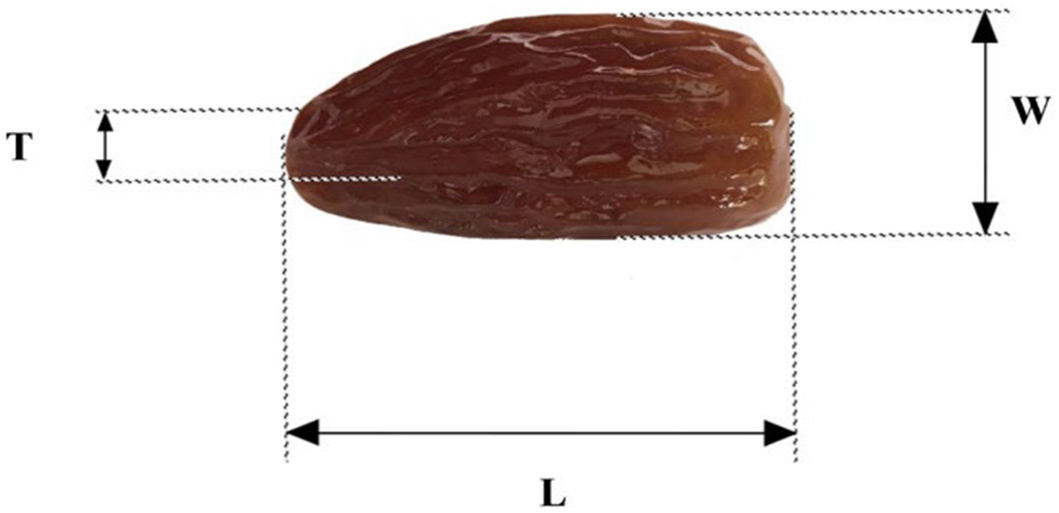
Dimensional characteristics of date fruit: *L*, length; *W*, width; *T*, thickness.

Experimentally obtained and/or statistically calculated linear dimensions play a crucial role in the development of effective drying and storage techniques (Desai et al., 2019). In this study, three key linear dimensions, length (*L*), width (*W*), and thickness (*T*), were experimentally determined, as depicted in Figure 2. Additionally, two other linear dimensions, arithmetic diameter (*D*_a_) and geometric mean diameter (*D*_g_), were calculated using the following equations (Desai et al., 2019; Djouab et al., 2016; Li et al., 2011):

(1) $$D_{a}=\frac{\left(L\cdot W\cdot T\right)}{3}$$

(2) Dg=L·W·T13

#### 2.2.2 Sphericity dimension

Determining the sphericity (*Φ*) dimension is crucial in designing handling equipment for date palm fruit. The calculation of sphericity values involves determining the triaxial ellipsoid with length, width, and thickness. This parameter is measured using the following equation (Desai et al., 2019; Jahromi et al., 2008):

(3) ∅=L·W·T13L=DgL

#### 2.2.3 Surface area

The surface area (*S*) of date fruit is a nonlinear dimension determined by analogy using the following equation (Desai et al., 2019; Li et al., 2011):

(4) S=πL·M·T23=π·Dg2

#### 2.2.4 Volumes and aspect ratio

As commonly known, date fruit volume is a crucial indicator influencing consumer acceptance (Sahin, 2003). This study investigates three types of volumes. The actual volume (*V*_m_) is measured using the water displacement method (Shahbazi & Rahmati, 2013). The two other theoretical volumes are calculated by assuming that the date fruit has a regular geometric shape as an oblate spheroid (*V*_osp_) and ellipsoid (*V*_ellip_). These calculated volumes are measured using the following equations (Thrall et al., 1978):

(5) $$V_{\mathrm{osp}}=\frac{4\pi }{3}\left(\frac{L}{2}\right){\left(\frac{W}{2}\right)}^{2}$$

(6) $$V_{\text{ellip}}=\frac{4\pi }{3}\left(\frac{L}{2}\right)\left(\frac{W}{2}\right)\left(\frac{T}{2}\right)$$

The aspect ratio (*R*_a_) is measured to indicate the tendency toward an oblong shape related to the larger two dimensions (*L* and *W*) with the following equation (Varnamkhasti et al., 2008):

(7) $$R_{a}=\frac{W}{L}\times 100$$

#### 2.2.5 Bulk and real density

Density (*ρ*) is experimentally determined using a calibrated graduated cylinder. The process involves shaking to ensure complete fitting, and then the content of the cylinder is weighed (Thrall et al., 1978). The density is calculated using the following equation (Desai et al., 2019):

(8) ρ=MV

Real density (*ρ*_t_) is determined using the actual volume, while theoretical density (*ρ*_b_) is determined using the calculated volume (Djouab et al., 2016). This parameter proves useful in the design of silos and storage bins (Varnamkhasti et al., 2008).

### 2.3 Mass modeling based on physical properties

From a consumer standpoint, date fruits with similar weight and symmetrical shapes are highly favored. Organizing dates by mass can help mitigate packaging and transportation expenses and result in an excellent packaging arrangement. In light of this, determining the co‐relationships between mass and linear and nonlinear dimensions proves to be beneficial and applicable (Khoshnam et al., 2007; Sudhir et al., 2019).

The appropriate model for predicting mass was investigated by fitting the obtained physical properties with linear (Equation 9), quadratic (Equation 10), S‐curve (Equation 11), and power (Equation 12) models. These models, measured on an industrial level, are applied using the following equations (Shahbazi & Rahmati, 2013; Sudhir et al., 2019), respectively:

(9) $$M=b_{0}+b_{1}\cdot X$$

(10) $$M=b_{0}+b_{1}\cdot X+b_{2}\cdot X^{2}$$

(11) $$M=b_{0}+\frac{b_{1}}{X}$$

(12) $$M=b_{0}\cdot X^{b_{1}}$$

In the equations, where *M* is the mass (g), *X* represents the value of the physical dimension affirming the relationship with the fruit mass, and *b*_0_, *b*_1_, and *b*_2_ are the curve‐fitted parameters. The statistically obtained mass models, along with well‐defined values for *b*_0_, *b*_1_, and *b*_2_, are determined by identifying the most suitable fit, which is achieved by selecting the model with the highest *R* ^2^ rate (closest to unity; Shahbazi & Rahmati, 2013).

The statistical methodologies applied in this study involved rigorous multivariate data analysis. Various mathematical models, including linear, quadratic, S‐curve, and power models, were meticulously employed to unravel intricate relationships between physical properties and masses of Algerian date fruits. Notably, these analyses were conducted using Microsoft Excel integrated with a Microsoft 365 subscription, selected for its computational prowess and widespread acceptance. This software suite provided a robust statistical framework, ensuring standardized, reproducible, and transparent analyses. The adoption of Microsoft Excel aligns with its versatility, enabling intricate calculations in accordance with the exacting standards of scientific inquiry, thereby enhancing the scholarly integrity of this research.

### 2.4 Mass model development using multivariate data analysis

In the study of date palm fruits, the mass is influenced by numerous factors, making multivariate data analysis (MVDA) an appropriate statistical approach for modeling. The primary objective of MVDA is to extract relevant information from large datasets. Buvé et al. (2022) extensively reviewed the application of MVDA in various food analyses for classification or prediction (Buvé et al., 2022), spanning diverse items such as cheese, sea cucumber, potato, strawberry jam, cereal flour, milk, fruit, and vegetables. MVDA has also been instrumental in revealing sensor data in several food applications (Abu‐Khalaf, 2021; Abu‐Khalaf et al., 2018; Najjar & Abu‐Khalaf, 2021).

In the context of this paper, MVDA was employed to test the mass modeling of the studied date palm fruits. The Unscrambler Software (10.3, Camo AS, Norway) was utilized to assess the correlation between mass and other parameters. For modeling the mass of both varieties alone and together, partial least squares (PLS) was employed. Here, mass served as the Y matrix, while other physical parameters constituted the X matrix, ensuring that all physical parameters were considered in the model. Cross‐validation with a random selection of 20 segments was employed. PLS, recognized as a robust method for regression, has proven effective in handling multiple correlation issues, even with relatively small sample sizes (Nie et al., 2023).

## 3 RESULTS AND DISCUSSION

### 3.1 Physical properties of date fruits

The physical properties of the examined date palm fruit varieties, Deglet Noor and Arechti, in Biskra region/Algeria have been comprehensively documented and are summarized in Table 1. The analysis of their linear dimensions reveals a significantly higher mean length in Deglet Noor (46.23 mm) compared to Arechti (43.24 mm), indicating a distinct disparity in the *L* value between the two varieties. In contrast, the remaining linear dimensions width (*W*), thickness (*T*), diameter perpendicular to the longitudinal axis (*D*_g_), and diameter parallel to the longitudinal axis (*D*_a_) display an inverse relationship, suggesting the elongated shape of Deglet Noor and the spherical shape of Arechti.

**TABLE 1:** Some physical properties of date palm fruits in Biskra region/Algeria (Deglet Noor/Arechti).

Variety	Deglet Noor				Arechti			
	Average	Maximum	Minimum	Significant level	Average	Maximum	Minimum	Significant level
*L* (mm)	46.23	59.28	38.04	*p* < .001	43.24	49.87	37.47	*p* < .001
*W* (mm)	21.98	25.58	16.75	*p* < .001	23.12	29.82	19.39	*p* < .001
*T* (mm)	12.29	20.50	8.03	*p* < .001	13.23	19.71	8.51	*p* < .001
*D*_g_ (mm)	23.14	27.45	19.86	*p* < .001	23.60	29.86	19.70	*p* < .001
*D*_a_ (mm)	26.83	30.93	23.12	*p* < .001	26.53	31.62	22.66	*p* < .001
Φ	0.50	0.57	0.41	*p* < .001	0.55	0.66	0.44	*p* < .001
*S* (mm^2^)	1685.79	2366.51	1238.07	*p* < .001	1755.35	2800.61	1219.06	*p* < .001
*V*_osp_ (mm^3^)	11,750.72	16,169.83	5585.33	*p* < .001	12,193.61	21,090.34	7927.41	*p* < .001
*V*_ellip_ (mm^3^)	6534.70	10,827.96	4097.36	*p* < .001	6960.69	13,939.99	4003.31	*p* < .001
*V* (mm^3^)	3980.91	6900.24	2448.00	*p* < .001	4377.54	9514.98	2473.20	*p* < .001
*M* (g)	14.63	17.14	10.92	*p* < .001	13.16	17.12	9.58	*p* < .001
*ρ*_b_ (g/mm^3^)	0.0037	0.0025	0.0045	*p* < .001	0.0030	0.0018	0.0039	*p* < .001
*R*_a_	47.54	43.15	44.03	*p* < .001	53.48	59.80	51.75	*p* < .001

The significant difference in weights between the two varieties is also apparent, with Arechti fruits exhibiting significantly lighter mean weights (13.16 g) compared to Deglet Noor (14.63 g). Moreover, both varieties demonstrate comparable maximum and minimum weights, primarily influenced by the geographical location of the plants (Zainal A'Bidin et al., 2020) and the characteristic traits shared by the semi‐dry category of date palm fruits.

Understanding the physical properties of date fruits is paramount, offering valuable insights into their morphological characteristics and inherent attributes. The comparative analysis conducted in this study between Deglet Noor and Arechti varieties reveals notable differences in their linear dimensions and weights, highlighting the necessity for a comprehensive assessment of these physical parameters. The distinct elongated shape of Deglet Noor and the spherical shape of Arechti hold significant implications for their visual appearance and potential applications across various culinary and industrial domains.

Furthermore, the observed variations in fruit weights provide insights into their density, directly impacting critical aspects such as handling, transportation, and storage considerations. The elucidation of the physical properties of date fruits facilitates the development of standardized grading systems, ensuring the supply of uniformly sized and shaped fruits that align with consumer preferences. These findings carry immense value for breeders, growers, and processors, empowering them to optimize production practices and enhance the competitive standing of the date industry in the global market.

### 3.2 Date fruit mass model based on physiological characteristics

#### 3.2.1 Modeling based on dimensions

To establish a reliable model for predicting the mass of date fruits, a comprehensive examination of their dimensional characteristics was undertaken, and the results are summarized in Table 2. It is evident that certain linear dimensions, specifically length (*L*), longitudinal diameter (*D*_a_), and transverse diameter (*D*_g_), exhibit the strongest correlation with fruit mass. These dimensions are crucial in grading and sizing date samples, widely recognized as essential physical parameters for these purposes (Ilić et al., 2019; Nunak & Suesut, 2007).

**TABLE 2:** The best models for predicting the mass of date palm fruits with the studied physical characteristics.

Dependent variable	Independent variable	Deglet Noor					Arechti				
		Best fitted model	*b*_0_	*b*_1_	*b*_2_	*R* ^2^	Best fitted model	*b*_0_	*b*_1_	*b*_2_	*R* ^2^
*M* (g)	*L* (mm)	S‐curve	25.306	−505.777	–	.5004	Quadratic	6.2073	0.0035	0.0036	.5219
*M* (g)	*W* (mm)	Quadratic	11.934	0.005	0.005	.2125	S‐curve	26.1232	−298.3130	–	.5468
*M* (g)	T (mm)	Quadratic	12.364	0.020	0.011	.3227	Power	6.7790	0.2574	–	.2915
*M* (g)	*D*_g_ (mm)	Quadratic	6.0527	0.0158	0.0146	.5511	S‐curve	27.8357	−344.6862	–	.5632
*M* (g)	*D*_a_ (mm)	Quadratic	4.4381	0.0134	0.0131	.6011	Power	0.1322	1.4030	–	.6382
*M* (g)	*Φ*	Quadratic	12.7806	3.4485	−0.8647	.2063	S‐curve	15.0209	−1.0192	–	.0825
*M* (g)	*S* (mm^2^)	Power	0.3474	0.500	–	.5439	Power	2.3897	0.2282	–	.5637
*M* (g)	*V*_osp_ (mm^3^)	Power	1.5217	0.2390	–	.3782	Power	1.0448	0.3027	–	.5322
*M* (g)	*V*_ellip_ (mm^3^)	Power	0.5564	0.3696	–	.5437	Power	0.2322	0.4295	–	.6219
*M* (g)	*V* (mm^3^)	Power	2.4266	0.2134	–	.5095	Power	0.6263	0.3446	–	.5622

The arithmetic and geometric mean diameters, in particular, serve as critical indicators for assessing fruit size and are integral to sorting and categorization processes. The findings of this study highlight *D*_a_ and *D*_g_ as the most appropriate parameters for accurately estimating the mass of the two examined types of date fruits. Accurate modeling of date fruit mass is paramount for effective grading and quality control within the date industry.

Establishing a clear relationship between the physical dimensions of the fruits and their mass provides valuable insights for the design of grading systems and the optimization of sorting procedures. The presented findings underscore the prominent role of specific linear dimensions, with particular emphasis on *D*_a_ and *D*_g_, in facilitating precise mass estimation for date fruits. These research outcomes contribute to the development of robust models that enable objective evaluation of fruit size, fostering informed decision‐making throughout various stages of handling, processing, and packaging.

By employing appropriate dimension‐based models, stakeholders within the date industry can enhance operational efficiency, improve product uniformity, and cater to the preferences and demands of consumers better.

#### 3.2.2 Modeling based on dimensions

The calibration and standardization of industrial processes related to fruit handling and processing place significant emphasis on verifying the sphericity of the fruit (Ilić et al., 2019). In this study, a thorough investigation was conducted to explore the relationship between the surface area of the analyzed fruits and their corresponding masses. The results demonstrate a noteworthy congruence between fruit mass and surface area, with the latter serving as a robust parameter for developing mass models.

However, it should be noted that the sphericity dimension, quantifying the deviation from a perfectly spherical shape, exhibited a relatively weaker fitting capacity within the mass modeling framework. Specifically, the quadratic model yielded an *R* ^2^ value of 0.2063 for Deglet Noor and 0.0825 for Arechti, while the S‐curve model achieved similar results. Accurate modeling of fruit mass is of utmost importance for ensuring precise calibration and optimization in diverse industrial processes. Within this context, the evaluation of fruit sphericity assumes significant relevance (Ilić et al., 2019).

In this investigation, a comprehensive analysis was carried out to examine the correlation between fruit mass and surface area, with the latter emerging as a particularly pertinent parameter for mass modeling. The findings reveal a strong alignment between fruit mass and surface area, underscoring the efficacy of this dimension in accurately predicting mass. However, it is imperative to acknowledge that the sphericity dimension, capturing the departure from an ideal spherical shape, demonstrated a relatively weaker association with the mass model. The quadratic and S‐curve models attained *R* ^2^ values of 0.2063 and 0.0825 for Deglet Noor and Arechti, respectively, indicating their limited fitting capabilities in this specific context.

#### 3.2.3 Modeling based on volume

The results reveal a notable correlation between volumetric measures and the corresponding masses of both Deglet Noor and Arechti varieties. The high degree of fitting observed between volume and mass underscores the efficacy of volumetric measurements in developing accurate mass models for these date palm fruits. The ellipsoid shape, a prominent characteristic of semi‐dry date palm fruits, provides an intriguing avenue for investigating the relationship between volume parameters and fruit mass.

In this study, careful attention was given to two volumetric indicators, namely V and Vellip, in relation to the mass of Deglet Noor and Arechti date palm fruits. Notably, the volumetric parameters exhibited a strong fitting capacity with fruit mass for both varieties. These findings highlight the significance of volumetric modeling in facilitating precise and reliable mass estimation for semi‐dry date palm fruits, contributing to enhanced efficiency and accuracy in various industrial applications.

### 3.3 Date fruit advanced mass model

**FIGURE 3:**
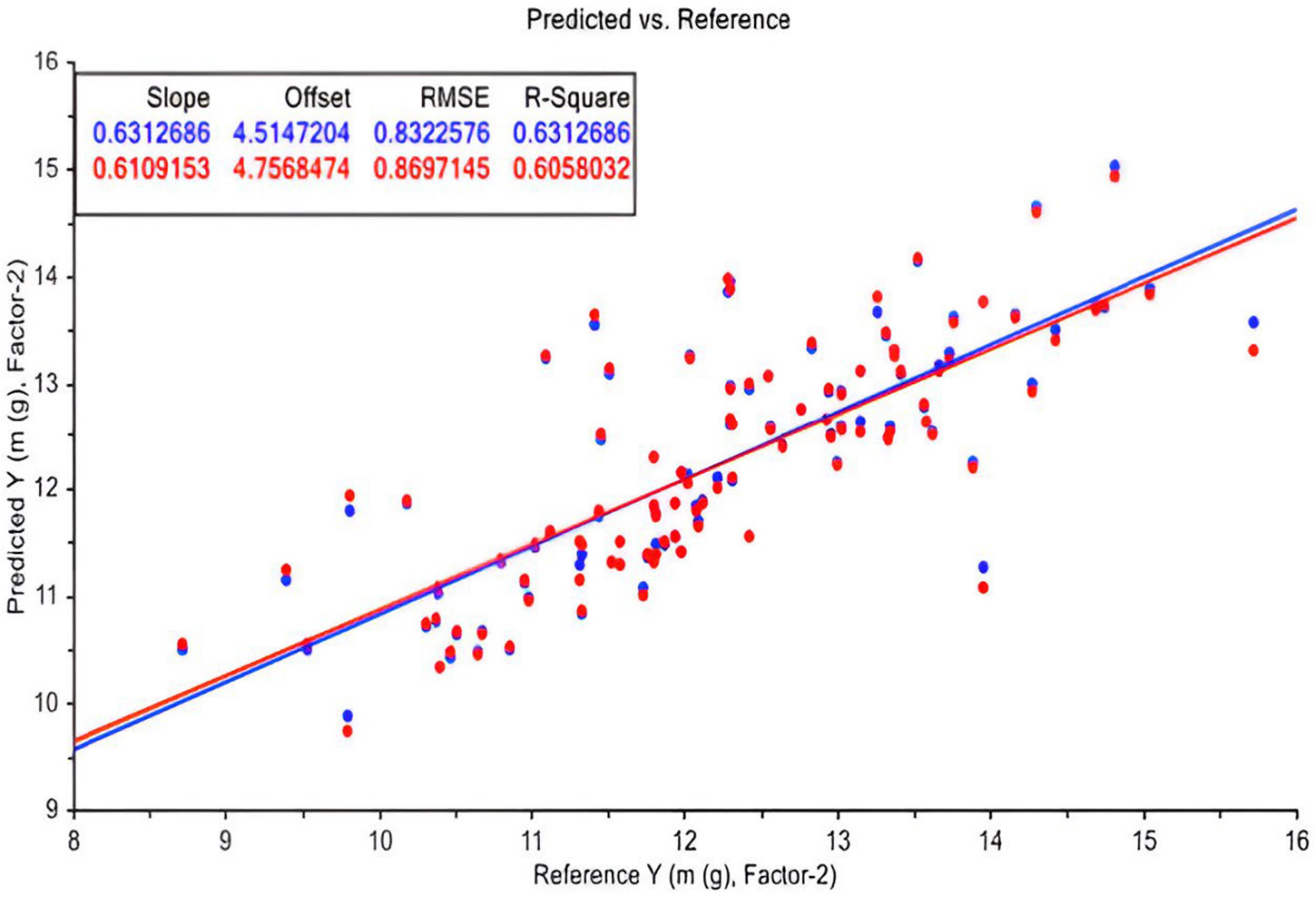
PLS model for Deglet Noor mass. Calibration (Blue (upper row) in the upper rectangular) and validation (Red (bottom row) in the upper rectangular) sets (some samples as outliers were taken out).

Figure 3 illustrates the predicted versus measured (reference) mass of Deglet Noor. Two principal components, explaining 79% and 63% of the variations in *X* (all parameters: volume and dimension) and *Y* (fruit mass), respectively, were utilized. The correlation of the model is approximately 0.6.

**FIGURE 4:**
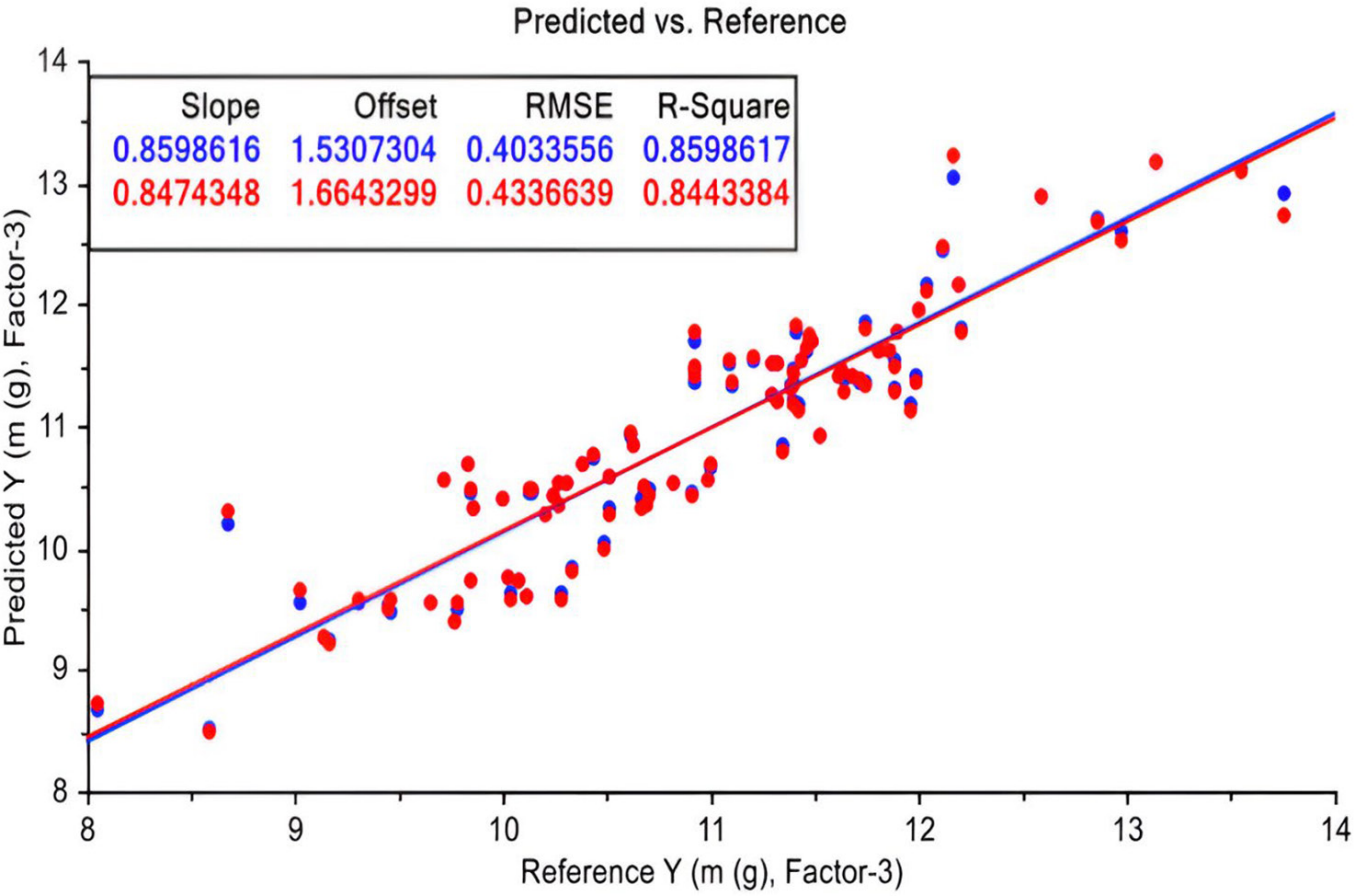
PLS model for Arechti mass. Calibration (Blue (upper row) in the upper rectangular and validation (Red (bottom row) in the upper rectangular) sets (some samples as outliers were taken out).

Figure 4 depicts the predicted versus measured (reference) mass of Arechti. Three principal components were employed, explaining 88% and 85% of the variations in *X* (all parameters: volume and dimension) and *Y* (fruit mass), respectively. The correlation of the model is approximately 0.84.

**FIGURE 5:**
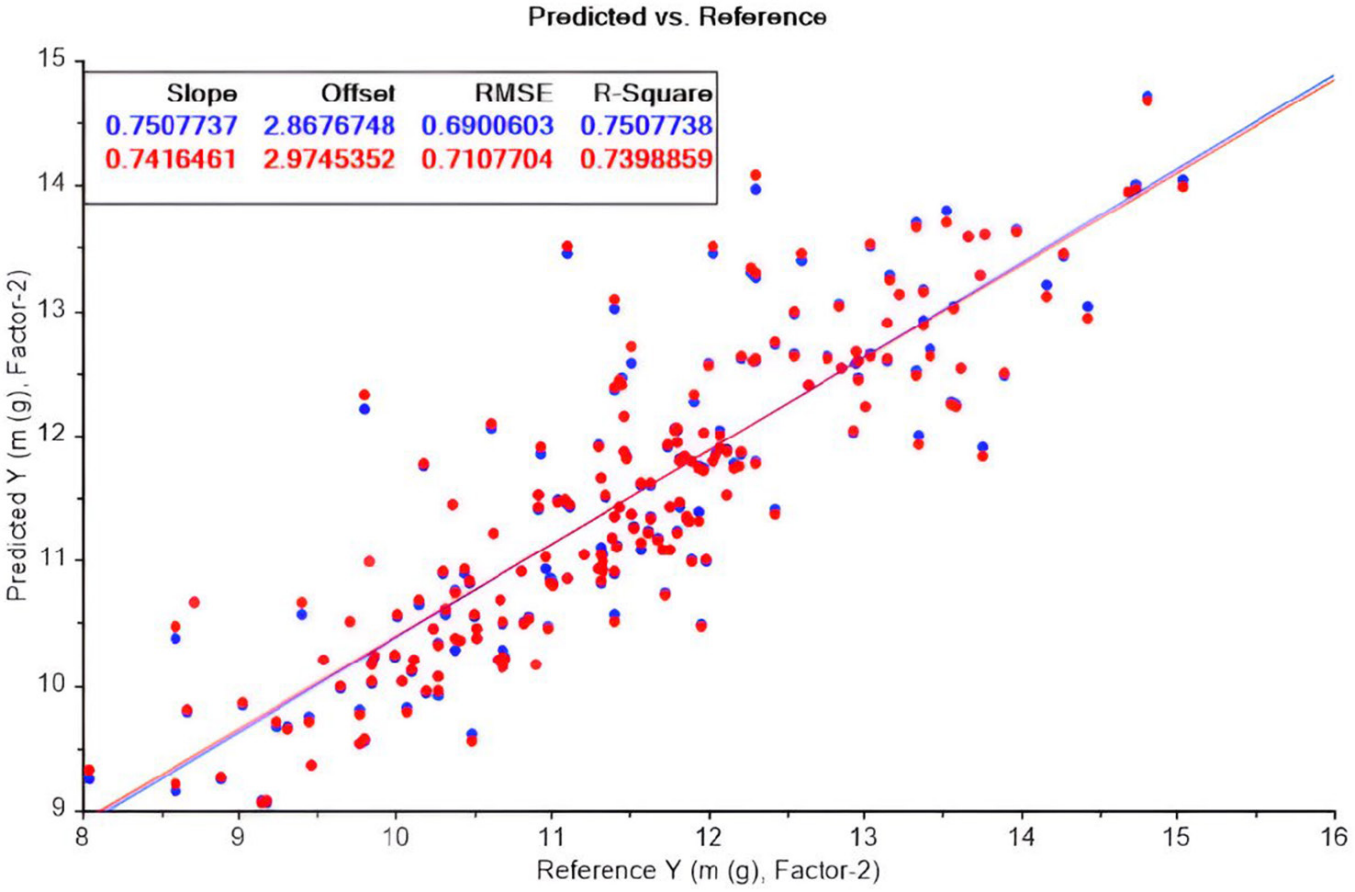
PLS model for both Deglet Noor and Arechti mass. Calibration (Blue (upper row) in the upper rectangular and validation (Red (bottom row) in the upper rectangular) sets (some samples as outliers were taken out).

Figure 5 illustrates the modeling of the mass of both varieties together in one model. The figure presents the predicted versus measured (reference) mass of the two varieties of fruit. Two principal components were utilized, explaining 79% and 75% of the variations in *X* (all parameters: volume and dimension) and *Y* (fruit mass), respectively. The correlation of the model is approximately 0.74. PLS successfully modeled the mass of both date varieties with a commendable correlation of 75%, demonstrating about 10% relative error (i.e., RMSE/Range).

## 4 CONCLUSION

In conclusion, this comprehensive study delved into the nuanced evaluation of the physical properties and mass models of two prominent semi‐dry Algerian date fruit varieties, Deglet Noor and Arechti. The meticulous analysis uncovered distinctive variations in linear dimensions and weight between these varieties. Notably, the arithmetic and geometric mean diameters emerged as pivotal parameters, demonstrating their efficacy in predicting the mass of these date fruits. The implications of these findings extend far beyond mere academic discourse. The insights gleaned from the study have the potential to revolutionize the date industry by informing the development of more efficient handling, sorting, processing, and packaging techniques. By strategically implementing a sizing and conserving system grounded in the identified physical properties, stakeholders stand to optimize their operational processes. This optimization, in turn, promises to reduce transportation costs and elevate consumer satisfaction through the consistent availability of uniformly sized and shaped date fruits. The successful application of multivariate data analysis (MVDA) in modeling the mass of each variety individually, as well as both varieties together, underscores the robustness of the developed models. This achievement not only enhances our understanding of these specific date varieties but also opens avenues for predicting mass using a broader spectrum of physical parameters. As the study draws to a close, it is evident that these research outcomes contribute not only to the academic realm but also hold immense practical relevance for industry professionals. Looking ahead, further research endeavors in this domain could expand our understanding to encompass a broader range of date fruit varieties. This expansion, coupled with the practical application of these findings, has the potential to propel the date industry toward greater efficiency, innovation, and consumer satisfaction.

## AUTHOR CONTRIBUTIONS

**Messaoud Roumani:** Data curation (lead); writing – original draft (equal). **Rania Remmani:** Investigation (equal); methodology (equal); resources (equal); software (equal); writing – original draft (equal); writing – review and editing (equal). **Malek Miladi:** Investigation (equal); methodology (equal); resources (equal); software (equal); writing – original draft (equal); writing – review and editing (equal). **Nawaf Abu‐Khalaf:** Data curation (equal); methodology (equal); software (equal); writing – review and editing (equal). **Antonio Ruiz Canales:** Funding acquisition (lead); writing – review and editing (equal).

## FUNDING INFORMATION

This project received support from Professor Antonio Ruiz Canales of the Department of Engineering at Miguel Hernández University in Alicante, Spain.

## CONFLICT OF INTEREST STATEMENT

All authors declare there is no conflict of interest.

## ETHICS STATEMENT

Not applicable.

## Data Availability

Research data associated with this manuscript are not made available for sharing.
